# Affordability of Essential Medicines and Associated Factors in Public Health Facilities of Jimma Zone, Southwest Ethiopia

**DOI:** 10.1155/2021/6640133

**Published:** 2021-03-16

**Authors:** Eyassu Mathewos Oridanigo, Waju Beyene Salgedo, Feyera Gebissa Kebene

**Affiliations:** ^1^Department of Nursing, College of Medical and Health Sciences, Wachemo University, Durame Campus, Durame, Ethiopia; ^2^Department of Health Economics, Policy and Management, Faculty of Public Health, Institute of Health, Jimma University, Jimma, Ethiopia; ^3^Department of Public Health, College of Medicine and Health Sciences, Ambo University, Ambo, Ethiopia

## Abstract

**Background:**

Affordability is one of the key dimensions for access to essential medicines, and poor affordability impedes access to treatment in health facilities. The concept of affordability is associated with the issue of impoverishment and catastrophic expenditure. The provision of affordable and appropriate essential medicines is a vital component of a well-functioning health system.

**Objective:**

The objective of this study was to assess the perceived affordability of essential medicines and associated factors in public health facilities of the Jimma Zone, Southwest Ethiopia.

**Methods:**

A facility-based cross-sectional study design was employed. The study was conducted from March 28 to April 30, 2018, in the public health facilities of Jimma Zone, Southwest Ethiopia. Based on the WHO operational package for assessing, monitoring, and evaluating a country's pharmaceutical situations, health facilities were selected from each selected district using lower-, middle-, and higher-level criteria, making a total of 30 health facilities. For the exit interview, the total sample size was proportionally allocated for each of the selected health facilities. The data from the patient exit interview were collected using interviewer-administered structured questionnaires. The data were checked for their completeness, edited, and coded. Following this, they were entered into EpiData 3.1 and exported to SPSS version 23 for analysis. Multivariable logistic regression analysis was performed using the backward LR method to identify factors independently associated with dependent variables.

**Result:**

Six hundred and six patients participated in the study with a response rate of 97%. Among the total patients, 63.9% characterized the prescribed medicines as not affordable. The level of the health facility [AOR (95% CI) = 3.848(2.144,6.905) and *p* ≤ 0.001], number of dispensed medicines [AOR (95% CI) = 0.326(0.215–0.493) and *p* ≤ 0.001], occupation [AOR (95% CI) = 3.354(1.793–6.274) and *p* ≤ 0.001], family income [AOR (95% CI) = 3.897(1.497–10.145) and *p*=0.005], place of residence [AOR (95% CI) = 2.100(1.331–3.315) and *p*=0.001] and number of economically dependent family members [AOR (95% CI) = 2.206(1.165–4.175) and *p*=0.015] were significantly associated with the perceived affordability of essential medicines.

**Conclusion:**

The average cost of dispensed medicines in the surveyed health facilities was not affordable for most of the patients. We recommend both social- and community-based health insurance schemes should be expanded to the study area.

## 1. Background

According to the World Health Organization (WHO), essential medicines are “those that satisfy the priority health-care needs of the population, and they are selected with due regard to public health relevance, evidence on efficacy and safety, and comparative cost-effectiveness” [1]. Affordability is one of the key dimensions for access to essential medicines, and poor affordability impedes access to treatment in health facilities [2]. The concept of affordability of medicines is related with the issue of catastrophic expenditure and impoverishment [3]. High medicine prices and low affordability are key impediments to access to treatment in many low- and middle-income countries. Certainly, in those countries where the majority of the population still buys medicines through out-of-pocket payments (OOP), the high cost of medicines (relative to the household budget) means that an illness in the family exposes the family to the risk of catastrophic expenditure [4].

Around the world, most leading causes of death such as malaria, infectious diseases, and HIV/AIDS can only be treated or prevented effectively by having appropriate medicines consistently available and affordable to people [5]. About 10 million lives a year could be saved by improving access to essential medicines [6].

One-third of the global population lacks regular access to needed medicines. The situation is even worse in the poorest countries of Africa and Asia, where as much as half of the population lacks such access [7]. The main reason for not obtaining life-saving medicines in these countries was lack of purchasing power [8]. In low- and middle-income countries where 71% of the world's population is living, medicines accounted for 11% of global pharmaceutical expenditure which is very low when compared to high-income countries, which accounted for 78.5% of global pharmaceutical expenditures [9].

The Ethiopian National Drug Policy aimed to ensure adequate supply of drugs required for treatment of diseases affecting the majority of the country's population [10]. To achieve this, the country developed a national essential drug list and recommends all public health facilities to prepare a facility list of drugs from the National Essential Drug list (EDL) to avail the most needed drugs at every level of healthcare at all times with affordable cost [11]. According to the national baseline study conducted on drug use indicators in the country, the percentage of drugs prescribed from the EDL was 99% [11], and the finding from multiple studies conducted in different parts of the country showed that almost all drugs were prescribed from the EDL [12–14].

The Ethiopian health sector is generally underfinanced by both global and regional standards, and it is hugely dependent on donors or the rest of the world and direct payment by households with OOP expenditure accounting for 49.9% and 33.7% of the national health expenditure, respectively [15]. Although efforts have been made to increase the accessibility of essential drugs (EDs) such as increasing budget allocation by the government of Ethiopia, researches showed that still the medicines were not affordable for the majority of the people, especially for the poor [16]. The main agency for procuring and distributing pharmaceuticals in the public sector is the Ethiopian Pharmaceuticals Supply Agency (EPSA), and it buys medicines through a tender system and then distributes them throughout the country to hospitals, health centers, health posts, and pharmacies [17]. The dispensing of drugs in Ethiopia is carried out through general pharmacies, hospital pharmacies (special, inpatient, and outpatient), drug stores, and rural drug vendors, and it is owned by public health facilities, city councils, private players, and the Red Cross Society [18]. Even though the Federal Ministry of Ethiopia (FMOE) has made tremendous efforts in stock management to supply safe and affordable drugs to public health facilities, the shortage of health financing affects the facilities with frequent drug shortages [19]. A survey conducted in pilot districts of the country showed that the overall enrolment of the community-based health insurance was 48% [20]. Moreover, the share of the employer-provided drug insurance was only 0.2% of the total drug expenditure in 2005-2006 [21]. For patients visiting health facilities, drugs can take up more than half of the actual cost of a visit that increases the chance of incurring catastrophic health expenditures and the associated risks of falling into poverty [22]. Therefore, this study was aimed to assess the perceived affordability of essential medicines and associated factors in public health facilities of the Jimma Zone, Southwest Ethiopia.

## 2. Methods

A facility-based cross-sectional study design was employed from March 28 to April 30, 2018, in public health facilities of Jimma Zone, Southwest Ethiopia. The Jimma Zone is one of the 22 zones in the Oromia region, and its administrative centre, the town of Jimma, is located 352 km southwest of Addis Ababa, the capital city of Ethiopia. The zone is divided into 20 districts and a two-town administration (Jimma and Agaro) with a total of 545 kebeles that cover a total area of 184,125.4 km^2^ with a total population of 3,345,112, among which 89.69% are rural inhabitants. In Jimma Zone, there were 1 tertiary hospital (Jimma University specialized and teaching hospital), 8 primary hospitals, 122 health centers, and 513 health posts.

The study population was all selected public health facilities that were found in five districts of the zone and sampled out patients who visited the selected public health facilities during the study period. Patients with fee waivers, who were few in number in the study area, were excluded from the study since they can obtain their prescribed medicines without payment and, thus, have no affordability problem. Program medicines and medicines for exempted services were also excluded from the study. The sample size for health facilities was determined based on the WHO's recommended operational package for assessing, monitoring, and evaluating countries' pharmaceutical situations [23]. Based on these recommendations, six health facilities were selected from each selected district using lower-, middle-, and higher-level criteria, making a total of 30 health facilities. Patients' sample size was calculated using a single population proportion formula for a cross-sectional study with the assumptions of a 95% confidence level, a 5% margin of error, and 44% of patients who were able to obtain their prescribed medicines with affordable prices in public health facilities in South Wollo, Ethiopia [24]. Since a multistage sampling technique was employed, a design effect of 1.5 was used to adjust for the sampling variability, i.e., to have the same sampling variance as compared to simple random sampling of the same sample size, and by considering a 10% nonresponse rate, the final sample size was *n* = 626.

The sampling procedure was also based on WHO recommendations, which were previously used for determining the sample size for health facilities. It recommends selecting five districts or geographical areas for assessing pharmaceuticals in public health facilities. Based on these recommendations, one capital city/town, one rural/remote district, and three other randomly selected middle-level districts among those remaining were included [23]. The town of Jimma was selected as the capital town or centre of the study area, while Nono Benja was selected as the most rural geographical area, and the three districts Mencho, Omo Nada, and Seka Chekorsa were selected randomly by lottery from the remaining districts. The same procedure was employed for selecting health facilities; one big health facility, one remote and small health facility, and four middle-level health facilities were included from each selected district of the zone. For patients' exit interviews, the total sample size was proportionally allocated for each of the selected health facilities based on their average daily number of outpatients calculated from the HMIS (Health Management Information System) monthly report for the same month in the last year prior to data collection. To obtain the average daily number of outpatients for each health facility, the last year's HMIS monthly report for outpatients of the same month with the data collection period was used and calculated as the number of patients visited the outpatient department of the facility in the same month divided by thirty. Finally, patients were interviewed consecutively while leaving the dispensing units (pharmacies).

Face-to-face patient exit interview data were collected by using interviewer-administered structured questionnaires focusing on patients' sociodemographic, socioeconomic and health-services-related information, and prescription papers of the interviewed patients were reviewed to obtain the relevant information on the perceived affordability of dispensed medicines and associated factors. For children less than 18 years of age, data were obtained from parents or guardians. The questionnaires were prepared by reviewing similar studies and were modified according to the local context [22, 24, 25]. The interview questions were arranged based on the panel experts' comments and suggestions. At this point of the process, a few amendments were made on the questions in terms of their content and structure. Six data collectors, who were diploma pharmacy technicians and two health officers, were recruited to collect the data and supervise the data collection process, respectively. The data collectors were selected from health centers outside of the study area in order to minimize interviewer bias, and they were selected based on the ability to speak both Affan Oromo and Amharic (the local languages). Two days of training on the aim of study and methods of data collection was given to them by the principal investigator, and a pretest was conducted outside the study area on 5% of the sample size to determine the clarity of the items and the consistency of the responses. Based on the study area's top 12 morbidities that can be treated as outpatient, the affordability of essential medicines based on the selected disease was assessed.

The affordability of the cost of a single course of treatment for 12 conditions was measured by comparing it with the daily wage of the Lowest-Paid Government Worker (LPGW). Standard treatments were understood as the full course of therapy for acute conditions or a one-month course of therapy for chronic conditions, in which therapy continues indefinitely [26]. The lists of key medicines was taken from the 2014 National Essential Medicines List of Ethiopia and National Standard Treatment Guideline to treat the most common health problems in the study area. The price data were collected from health facilities by reviewing the most recent price data that were recorded in the health facility and by asking a store keeper or a person in charge of the pharmacy in medicine outlets for medicines which have no stock records.

The collected data were entered, cleaned, and analyzed using Statistical Package of Social Sciences (SPSS) version 23. Data for the affordability of the selected essential medicines were entered and analyzed using Microsoft Excel 2007. Complete case analysis was conducted since there was no missing data. Descriptive statistics were computed, and tables, graphs, and numerical summaries were created to present the results. A chi-square test was conducted, and all variables were checked by cross tabulation for fulfilling chi-squared test assumptions of 80% expected frequency greater than five and all cells' expected frequency greater than one. Bivariate analysis was used to identify factors associated with the perceived affordability of essential medicines, and variables with a *p* value of <0.25 in bivariate analysis were considered as candidates for multiple logistic regressions. Multiple logistic regressions were performed using the backward LR method to identify factors independently associated with dependent variables. The degree of association between dependent variables and independent variables was measured using an Adjusted Odds Ratio (AOR) with 95% Confidence Interval (CI) at a significance level of ≤0.05. Hosmer–Lemeshow goodness-of-fit statistics was used to check the goodness of fit of the model with a *p* value of 10%.

### 2.1. Operational Definitions

**Perceived affordability:** it is patients' perception on cost of dispensed medicines based on their ability and prices of medicines for diagnosed diseases, and it is classified as affordable if the patients were able to obtain the dispensed medicines and not affordable if they were unable to obtain it with a minimum price.

**Affordability based on selected diseases:** medicine that costs the equivalent to one day's salary of the LPGW to purchase a full course of treatment for an acute condition or a one-month course of treatment for a chronic condition was generally considered as affordable, and treatment that costs more than this was considered as unaffordable [26].

At the time of the survey, the lowest-paid unskilled government worker earned 28.67 ETB (US$ 1.05) per day with a currency exchange rate of 1 US$ = 27.29 ETB.

**Days wages** **=** median medicine price found in the facilities times the number of units in a standard course of treatment/daily salary of an LPGW [23].

**Economically dependent family member**: any family member (relative or nonrelative) who relies on the head of the household for financial support to purchase the prescribed medicines.

## 3. Results

### 3.1. Sociodemographic and Socioeconomic Characteristics of Respondents

In this study, out of 626 patients sampled, 606 responded to the questionnaires, making a response rate of 97%. Out of the patients, 324 (53.5%) were males. About half of them, 307 (50.7%), were married, while only 37 (6.1%) were divorced. Regarding the residential area, majority of them, 390 (64.4%), were rural dwellers. Moreover, 221 (34.8%) of them had more than four family members being economically dependent (Table 1).

### 3.2. Health-Facility- and Health-Service-Related Characteristics

Among the total patients who visited the surveyed health facilities, 438 (72.3%) and 168 (27.7%) participated from the health centers and hospitals, respectively. Regarding the number of dispensed medicines per patient, on average, 2.46 medicines were dispensed. 51.2% of patients obtained less than or equal to two dispensed medicines per prescription while the remaining, 48.8%, obtained more than two medicines per prescription.

### 3.3. Affordability of Essential Medicines Based on Selected Diseases

Affordability of generic medicines in the surveyed health facilities was varied for most conditions with standard treatment costing a day's wage or less of the LPGW. The average minimum days' wage for 12 standard treatments was 1.02 (ranging from 0.42 to 3.66). Treatments costing over one day's wage of the LPGW were bronchial asthma, gardiasis, skin and subcutaneous tissue infection, and typhoid fever. Treatments costing less than one day's wage of the LPGW were adult and pediatric pneumonia, diabetic mellitus (DM), hypertension, arthritis, peptic ulcer disease (PUD), and urinary tract infection (UTI). On average, an LPGW would need to work for 0.7 days in order to purchase a course of LPG (Lowest-Priced Generic) amoxicillin capsule to treat adult pneumonia. A course of LPG cotrimoxazole suspension costs 0.5 day's salary of the LPGW to treat child pneumonia. For the treatment of bronchial asthma, the LPGW would need to work 1.5 days to buy the LPG salbutamol inhaler in the surveyed health facilities (Table 2).

### 3.4. Affordability of Dispensed Medicines in Days Wages of an LPGW

**Figure 1:**
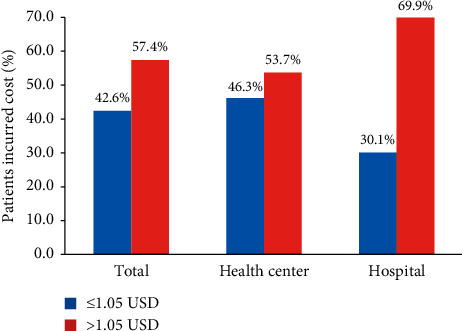
Percentage of patients who incurred costs on medicines in relation to the daily wage of an LPGW (1.05 USD).

On the exit interview of patients, who visited the surveyed health facilities, the total mean healthcare expenditure per patient was US$ 3.22 (3.07 days' wage) including transport cost. Patients spent, on average, US$ 2.32 (2.21 days' wage) on medicines, while the median expenditure was US$ 2.13 (2.02 day's wage). As a result, medicines accounted for 71% of the total health-care expenditure on visit. Among the patients for whom medicines were dispensed, 367 (60.6%) incurred costs more than US$ 1.05 (one day's lowest paid government worker salary). Based on health facility categories, 95 (69.9%) and 246 (53.7%) patients in hospitals and health centers incurred costs on medicines above the cut-off point, US$ 1.05 respectively (Figure 1).

### 3.5. Affordability of Essential Medicines Based on Consumers' Perception

From the total patients, 471(77.7%) and 135(22.3%) bought medicines from the public health facilities and outlets fully and partially, respectively. Of those patients who obtained medicines partially, 92.3% of them went to buy the remaining medicines from private sectors while 2.2% and 5.5% went to traditional medicines and their homes with partial treatment, respectively. Unavailability and the price of medicines were the main reasons for the inability to obtain the prescribed medicines from the surveyed health facilities. Based on their perception, 219 (36.1%) patients characterized the prescribed medicines as affordable.

### 3.6. Factors Associated with Perceived Affordability of Essential Medicines

Among eleven variables in bivariate analysis, nine of them had a *p* value of less than 0.25; hence, they were candidates for multivariable logistic regression analysis. Bivariate analysis revealed that the level of the health facility, number of dispensed medicines, income, place of residence, marital status, educational status, age, occupation, number of economically dependent family members were candidates for multivariable analysis (Table 3).

Those variables with a *p* value of less than 0.25 were again entered into multiple logistic regression models to obtain variables which were independently associated with an outcome variable, perceived affordability of essential medicines. The variables with a *p* value of less than 0.05 in multivariable logistic regression analysis were taken as significant predictors of the outcome variable.

The final model showed that there was a statistically significant association between health facility categories and perceived affordability of dispensed medicines (*p* value< 0.001), so that those patients who attended health centers were 3.848 times more likely to obtain the dispensed medicines with affordable prices than those who attended hospitals [OR (95% CI) = 3.848(2.144,6.905)].

In this study, the patients who had more than two medicines per prescription were 67% less likely to obtain the dispensed medicines with affordable prices than those who had less than or equal to two medicines per prescription [OR (95% CI) = 0.326(0.215, 0.493)] (Table 4).

## 4. Discussion

The affordability of a single course of treatment for selected diseases is calculated in terms of the lowest paid unskilled government worker's daily wage assuming that the income level of most of the poor is equivalent to the LPGW salary per individual level [27]. The results showed that the treatments for bronchial asthma and adult and child pneumonia were more costly than indicated by a study conducted in Kenya [27]. The variation might be due to the decreased median price of the medicines in health facilities at the time of data collection and the higher daily salary of the LPGW which was approximately US$ 3 as found in a previous study. The absence of a clear medicine pricing policy, high retail markups, and high variation in prices of medicines and the absence of a system of pharmaceutical evaluation influenced to raise pricing of drugs in the health facilities of Ethiopia [28].

In this study, most figures showed that the selected medicines were not affordable for common illnesses in the surveyed health facilities. This is because a single medicine was assessed, and a high salary was used compared to that reported in other studies in calculations of affordability. While affordability was measured in terms of only a single medicine, this may be insufficient in reality since most conditions are treated with more than one medicine. This observation was confirmed by this study, which found that the average number of the medicines per prescription was 2.71.

In this study, patients spent an average of US$ 2.32 (2.21 days' wage) on medicine through out-of-pocket (OOP), which is high as compared to the expenditure indicated by a study conducted in South Wollo, Ethiopia [24]. The mean variation might arise due to the presence of higher number of dispensed medicines per encounter in this study than the previous study. In this study, the average number of medicines per prescription was 2.71 compared to 1.72 in a previous study. When we compare this number with the salary of an LPGW, a patient should work more than two days in order to purchase prescribed medicines. The catastrophic health expenditure and the associated risk of falling into poverty are a direct result of the high cost of medicines [22].

In this study, 63.9% of the patients characterized dispensed medicines as not affordable. This finding is higher compared to the finding from the study conducted in the Jimma Health Centre which reported 47.8% [25]. The mean variation might arise due to the classification of a different level of the outcome variable in both studies. The outcome variable had three levels in a previous study, while it had two levels in this study. Patients may skip the prescribed medicines due to their high cost, and not taking medications as prescribed can cause serious health problems and unnecessary complications related to the medical condition [29].

This study found that the number of dispensed medicines was one of the inhibiting factors of the perceived affordability of dispensed medicines in the sampled health facilities. Patients who got more than two medicines per prescription were less likely to obtain the dispensed medicines with affordable prices than patients who got less than or equal to two medicines. The finding of this study is consistent with those of the previous study conducted in South Wollo, which found a negative association [24]. When the number of prescribed medicines increases per prescription, it raises the cost of medicines; hence, patients are less likely to get medicines from the visited health facilities, and increased medicine expenditures raise total health expenditures, thus bringing an economic burden on the patients [30].

The findings of this study also revealed that patients from health centers were more likely to receive dispensed medicines with affordable prices than patients from hospitals. This finding is supported by a study conducted in Ethiopia in 2015 [24]. This is confirmed by the finding from this study that 69.9% and 53.7% of patients visiting hospitals and health centers, respectively, incurred costs of medicines above the daily wage of an LPGW ($1.05). Hospitals prescribe medicines to treat chronic diseases for longer periods than health centers, which may be more expensive than medicines prescribed to treat diseases for short periods in health centers.

Occupational status was another important predictor of the perceived affordability of essential medicines. This study has shown that government employees and merchants were more likely to obtain the prescribed medicines with affordable prices than farmers. This finding is in line with findings from a study in Nigeria, which has also suggested that an individual's employment status has an effect on his or her ability to pay for dispensed medicine, and those with paid employment were more likely to afford medicines than self-employed people, and being employed (fulltime or part-time) tends to be associated with financial security [31]. In this study, family income was found to be a significant predictor of perceived affordability of dispensed medicines. Patients with a family income between US$171 and US$225 were more likely to obtain medicines with affordable prices than patients with a family income between US$10 and US$50. A similar study in the Jimma health centre showed that family income was a predictor of the perceived affordability of essential medicines [25]. It was also supported by another study conducted in West Nigeria [31]. The findings of this study showed that patients who came from urban areas were more likely to obtain medicines with affordable prices than patients who came from rural areas. This might be because people living in urban areas have higher sources of income than people living in rural areas. This was confirmed by the finding from this study, which found that the mean income of people who came from an urban area is US$ 88.6, which was higher than the mean income of people who came from rural areas, US$ 51.7.

## 5. Strengths and Limitations

The limitation of this study is that the minimum wage used for the calculation of affordability for standard treatment or individual medicines for some disease conditions is that from the formal sector, the LPGW. However, a substantial proportion of the patients in the study area may earn less than this amount, and a number of dependents may live on the wage of one person. Due to the cross-sectional study design, no causal inferences can be made regarding the temporal association between the potential factors and affordability. It is likely that recall or social desirability bias might occur during interview, and it might underestimate or overestimate the true effect or association.

Strength of this study is the use of guideline, the WHO operational package for assessing, monitoring, and evaluating countries' pharmaceutical situations. In addition, the study includes adequate sample size. Therefore, the findings from this study can be generalized to other populations.

## 6. Conclusions

Selected key essential medicines used for the treatment of common health problems were not affordable in the surveyed health facilities. The cost of dispensed medicines was not affordable for nearly two-thirds of the patients visiting the surveyed health facilities during the study period. The number of dispensed medicines was negatively associated, while the level of the health facility, occupation, family income, residence, and number of economically dependent family members were positively associated with the perceived affordability of essential medicines. District health offices and zonal health departments should expand both social- and community-based health insurance schemes to the study area to minimize out-of-pocket payment by patients; healthcare providers should improve their pattern of prescribing of medicines to minimize unnecessary cost paid for medicines by patients. All households should be enrolled in community-based health insurance schemes to minimize health expenditure and out-of pocket payments.

## Figures and Tables

**Table 1:** Sociodemographic and socioeconomic characteristics of patients in Jimma Zone public health facilities, May 2018.

S/N	Sociodemographic factors	Category of characteristics	Frequency (%)
1	Age	18–29	178 (29.4)
		30–44	249 (41.1)
		45–64	123 (20.3)
		≥65	56 (9.2)

2	Sex	Male	324 (53.5)
		Female	282 (46.5)

3	Residence	Urban	216 (35.6)
		Rural	390 (64.4)

4	Occupation	Farmer	154 (25.4)
		Merchant	135 (22.3)
		Student	70 (11.6)
		House wife	101 (16.7)
		Government employee	72 (11.9)
		Daily laborer	63 (10.4)
		Others^*∗*^	11 (1.8)

5	Marital status	Single	202 (33.3)
		Married	307 (50.7)
		Divorced	37 (6.1)
		Widowed	60 (9.9)

6	Monthly income	(10–50) $	277 (45.7)
		(51–115) $	206 (34.0)
		(116–170) $	68 (11.2)
		(171–225) $	40 (6.6)
		>225 $	15 (2.5)

7	Education	Illiterate	225 (37.1)
		Grade 1–8	156 (25.7)
		Grade 9–12	136 (22.4)
		Collage and above	89 (14.7)

8	Number of economically dependent family members	None	77 (12.7)
		One	81 (13.4)
		Two	85 (14)
		Three	81 (13.4)
		Four	71 (11.7)
		More than four	211 (34.8)

^*∗*^Others indicating nongovernmental and private employees.

**Table 2:** Affordability of a single course of treatment in days' wage of an LPGW for some disease conditions in Jimma Zone public health facilities, May 2018.

S/N	Disease conditions	Drug name, strength, and dosage forms	Standard Rx schedule	Affordability in days of wage
1	Adult pneumonia	Amoxicillin 500 mg cap	1 caps x3/day x7 = 21 caps	0.69
2	Pediatric pneumonia	Cotrimoxazole 240 mg/5 ml suspension	5 ml x 2/day for 7 days = 70 ml	0.51
3	Bronchial asthma	Salbutamol 0.1 mg/dose	1 inhaler of 200 doses	1.50
4	Diabetic mellitus	Glibenclamide 5 mg cap	1 tab x 2/day for 30 days = 60 tabs	0.56
5	Hypertension	Hydrochlorothiazide 25 mg tab	1 tab x1/day x30 = 30 tabs	0.93
6	Gardiasis	Metronidazole 250 mg cap	2 caps x 3/day x 7 days = 42 caps	1.05
7	Arthritis	Diclofenac 50 mg tab	1 tab x 2/day for 30 days = 60 tabs	0.42
8	PUD	Omeprazole 20 mg cap	1 cap x1/day for 30 days = 30 caps	0.52
9	UTI	Norfloxacillin 400 mg tab	1 tab x2/day x7 = 14 tabs	0.56
		Cotrimoxazole 480 mg tab	2 tabs x2/day x7 = 28 tabs	0.91
10	Skin and subcutaneous tissue infection	Cloxacillin 250 mg tab	2 caps x4/day x7 = 56 caps	1.19
11	Helminthiasis	Albendazole 400 mg tab	1 tab stat	0.45
12	Typhoid fever	Ciprofloxacillin 500 mg tab	1 tab x2/day x7 = 14 tabs	0.56
		Ceftriaxone 1 gm Iv, vial	1 vial x1/day x 7 = 7 vials	3.66
		Chloramphenicol 250 mg cap	2 caps x4/day x7 = 56 caps	1.74
Average days' wage				1.02

**Table 3:** Bivariate analysis of factors associated with perceived affordability of essential medicines in public health facilities of the Jimma Zone, Southwest Ethiopia.

Variable		Not affordable	Affordable	*p* value	95% CI for COR
Name	Category	N (%)	N (%)		
Age	18–29	117 (65.7%)	61 (34.3%)	0.397	(0.793,3.086)
	30–44	154 (61.8%)	95 (38.2%)	0.066^*∗∗*^	(1.260,3.569)
	45–64	74 (39.8%)	49 (60.2%)	0.056^*∗∗*^	(1.382,4.018)
	≥65	42 (75%)	14 (25%)		1

Marital status	Single	138 (68.3%)	64 (31.7%)	0.048^*∗∗*^	(1.008,4.236)
	Married	168 (54.7%)	139 (45.3%)	0.000^*∗∗*^	(1.846,7.359)
	Divorced	32 (86.5%)	5 (13.5%)	0.536	(0.221,2.192)
	Widowed	49 (81.7%)	11 (18.3%)		1

Sex	Female	183 (64.9%)	99 (35.1%)	0.622	1
	Male	204 (63%)	120 (37%)		(0.780,1.516)

Educational status	No education	166 (73.8%)	59 (26.2%)	0.303	1
	Primary	103 (66%)	53 (34%)	0.124^*∗∗*^	(0.928,2.259)
	Secondary	90 (66.2%)	46 (33.8%)	0.000^*∗∗*^	(1.015,2.285)
	College and above	28 (31.5%)	61 (68.5%)		(3.583,10.487)

Occupational status	Farmer	120 (77.9%)	34 (22.1%)	0.000^*∗∗*^	1
	Merchant	50 (37.0%)	85 (63.0%)	0.551	(3.579,10.059)
	Student	52 (74.3%)	18 (25.7%)	0.956	(0.633,2.358)
	House wife	79 (78.2%)	22 (21.8%)	0.000^*∗∗*^	(0.536,1.803)
	Government employee	23 (31.9%)	49 (68.1%)	0.195^*∗∗*^	(4.025,14.045)
	Daily laborer	54 (85.7%)	9 (14.3%)	0.763	(0.164,0.912)
	Others+	9 (81.8%)	2 (18.2%)		(0.162,3.803)

Place of residence	Rural	285 (73.1%)	105 (26.9%)	0.000^*∗∗*^	1
	Urban	102 (47.3%)	114 (52.7%)		(2.141,4.298)

Number of economically dependent family members	None	55 (71.4%)	22 (28.6%)	0.398	(0.716,2.318)
	One	42 (51.9%)	39 (48.1%)	0.000^*∗∗*^	(1.744,5.126)
	Two	44 (51.8%)	41 (48.2%)	0.000^*∗∗*^	(1.765,5.101)
	Three	44 (54.3%)	37 (45.7%)	0.000^*∗∗*^	(1.578,4.647)
	Four	41 (57.7%)	30 (42.3%)	0.003^*∗∗*^	(1.335,4.157)
	More than four	161 (76.3%)	50 (23.7%)		1

Estimated monthly income in USD	10–50$	210 (75.8%)	67 (24.2%)	0.007^*∗∗*^	1
	51–115$	133 (64.6%)	73 (35.4%)	0.000^*∗∗*^	(1.157, 2.557)
	116–170$	21 (30.9%)	47 (69.1%)	0.000^*∗∗*^	(3.915,12.571)
	171–225$	15 (40.0%)	25 (62.5%)	0.060^*∗∗*^	(2.603,10.485)
	>225$	8 (53.3%)	7 (46.7%)		(1.959,7.845)

Level of the health facility	Hospital	108 (78.3%)	29 (21.7%)	0.000^*∗∗*^	1
	HC	279 (59.5%)	190 (40.5%)		(1.618,3.975)

Number of dispensed medicines per patient	≤2	153 (49.4%)	157 (50.6%)	0.000^*∗∗*^	1
	>2	234 (79.1%)	62 (20.9%)		(0.181,0.369)

Length of Rx for diagnosed diseases	Rx requires ≥1 month	65 (68.4%)	30 (31.6%)	0.314	1
	Rx requires <1 month	322 (63%)	189 (37%)		(0.796,2.032)

^*∗∗*^Candidate variables with *p* < 0.25. +Others like private employees and NGO employees.

**Table 4:** Multivariable logistic regression analysis of factors associated with perceived affordability of essential medicine in public health facilities of the Jimma Zone.

Variables		Not affordable	Affordable	*p* value	AOR (95% CI)
Name	Category	N (%)	N (%)		
Level of the health facility	Hospital	108 (78.3%)	29 (21.7%)	—	1
	HC	279 (59.5%)	190 (40.5%)	0.000^*∗*^	**3.848** (2.144,6.905)^*∗*^

Number of dispensed medicines	≤2	153 (49.4%)	157 (50.6%)	—	1
	>2	234 (79.1%)	62 (20.9%)	0.000^*∗*^	**0.326** (0.215,0.493)^*∗*^

Place of residence	Rural	285 (73.1%)	105 (26.9%)	—	1
	Urban	102 (47.3%)	114 (52.7%)	0.001	**2.100** (1.331, 3.315)^*∗*^

Estimated monthly income in USD	10–50$	210 (75.8%)	67 (24.2%)	—	1
	51–115$	133 (64.6%)	73 (35.4%)	0.491	1.195 (0.720, 1.984)
	116–170$	21 (30.9%)	47 (69.1%)	0.053	2.149 (0.990,4.663)
	171–225$	15 (40.0%)	25 (62.5%)	0.005	**3.897** (1.497,10.145)^*∗*^
	>225$	8 (53.3%)	7 (46.7%)	0.912	0.930 (0.260,3.333)

Occupational status	Farmer	120 (77.9%)	34 (22.1%)	—	1
	Merchant	50 (37.0%)	85 (63.0%)	0.000^*∗*^	**3.354** (1.793,6.274)^*∗*^
	Student	52 (74.3%)	18 (25.7%)	0.671	1.171 (0.565,2.429)
	House wife	79 (78.2%)	22 (21.8%)	0.549	0.818 (0.424,1.578)
	Government employee	23 (31.9%)	49 (68.1%)	0.001	**3.686** (1.701,7.986)^*∗*^
	Daily laborer	54 (85.7%)	9 (14.3%)	0.015	**0.333** (0.138,0.806)^*∗*^
	Others	9 (81.8%)	2 (18.2%)	0.215	0.332 (0.058,1.898)

Number of economically dependent family members	None	55 (71.4%)	22 (28.6%)	0.854	0.935 (0.459, 1.908)
	One	42 (51.9%)	39 (48.1%)	0.008	**2.443** (1.259, 4.741)^*∗*^
	Two	44 (51.8%)	41 (48.2%)	0.015	**2.206** (1.165, 4.175)^*∗*^
	Three	44 (54.3%)	37 (45.7%)	0.029	**2.053** (1.075, 3.923)^*∗*^
	Four	41 (57.7%)	30 (42.3%)	0.237	1.510 (0.763, 2.990)
	More than four	161 (76.3%)	50 (23.7%)		1

AOR = adjusted odds ratio, CI = confidence interval, 1 = reference group. ^*∗*^Statistically significant at *p* < 0.05, Hosmer and Lomeshow test *p*=0.407, classification power = 77.6%.

## Data Availability

Data can be made available upon reasonable request from the corresponding author.
